# Fear no colors? Observer clothing color influences lizard escape behavior

**DOI:** 10.1371/journal.pone.0182146

**Published:** 2017-08-09

**Authors:** Breanna J. Putman, Jonathan P. Drury, Daniel T. Blumstein, Gregory B. Pauly

**Affiliations:** 1 Department of Ecology and Evolutionary Biology, University of California, Los Angeles, California, United States of America; 2 Section of Herpetology and Urban Nature Research Center, Natural History Museum of Los Angeles County, Los Angeles, California, United States of America; Estacion Experimental de Zonas Aridas, SPAIN

## Abstract

Animals often view humans as predators, leading to alterations in their behavior. Even nuanced aspects of human activity like clothing color affect animal behavior, but we lack an understanding of when and where such effects will occur. The species confidence hypothesis posits that birds are attracted to colors found on their bodies and repelled by non-body colors. Here, we extend this hypothesis taxonomically and conceptually to test whether this pattern is applicable in a non-avian reptile and to suggest that species should respond less fearfully to their sexually-selected signaling color. Responses to clothing color could also be impacted by habituation to humans, so we examine whether behavior varied between areas with low and high human activity. We quantified the effects of four T-shirt colors on flight initiation distances (FID) and on the ease of capture in western fence lizards (*Sceloporus occidentalis*), and we accounted for detectability against the background environment. We found no differences in lizard behavior between sites. However, lizards tolerated the closest approaches and were most likely to be captured when approached with the T-shirt that resembled their sexually-selected signaling color. Because changes in individual behavior affect fitness, choice of clothing color by people, including tourists, hikers, and researchers, could impact wildlife populations and research outcomes.

## Introduction

Increasing human populations threaten wildlife worldwide, not only by altering habitats, but also inducing changes to animal behavior [1–3]. Animals may perceive humans as predators [4], and this can indirectly alter their activity budgets and/or create physiological stress [5–7]. Animals are sensitive to varying degrees of human activity. Even small indirect disturbances by humans can contribute to cumulative negative impacts on wildlife populations because changes in individual behavior may affect fitness [8,9]. Nuanced aspects of human behavior, such as gaze direction [10,11], body posture [12], and familiarity or predictability [10,13,14] have been shown to influence animal behavior. Even the shutter noises from cameras modify behavior [15]. Given these challenges, animals should habituate by reducing responses over repeated encounters [16], and such habituation can reduce the loss of body condition in high human activity areas [17].

Previous studies have found that animals are sometimes, but not always, behaviorally affected by human clothing color. Studies on birds have found that flight initiation distances vary with observer clothing color [18–20]. Yet, other studies on a mammal (*Rupicapra rupicapra*) and a lizard (*Podarcis lilfordi*) found no differences in flight responses to different colored clothes [21,22]. The study on the mammal was conducted in a tourist location and habituation to humans could have reduced responsiveness to human activity, leading to little discrimination among clothing colors. Moreover, assessing general patterns is not currently possible because this research has not been guided by strong a priori hypotheses or study designs; for instance, all but one study on birds test behavioral differences between observers wearing bright orange versus dark gray, making it impossible to decouple the effects of color from detectability against the background environment. It is imperative that we develop a more predictive understanding of the effects of color on wildlife because such effects could impact research outcomes, conservation outcomes, and ecotourist experiences.

We suggest that the species confidence hypothesis could help explain the influence of clothing color on animal behavior. This hypothesis argues that birds should bias their attention toward colors in their environment that they are familiar with (i.e. the colors found on their own bodies), and they should be repelled by non-body colors [23,24]. This pattern has been found in multiple bird species, and in terms of flight responses, species with orange/red body patches are generally more tolerant of humans wearing orange or red compared to species that lack these colors [18–20,25]. Unfortunately, it is often impossible to determine whether birds are responding to a specific color, as mentioned above, and the taxonomic generality of the species confidence hypothesis remains uncertain. Previous studies on non-avian taxa (*R*. *rupicapra* [21] and *P*. *lilfordi* [22]) have so far been insufficient because either the study species lacked colored body patches or the researchers did not select T-shirts based on the species’ body color(s).

Here, we extend the species confidence hypothesis taxonomically by examining a non-avian reptile and conceptually by focusing on sexually-selected signaling colors. Because animal sensory mechanisms have evolved in part to be attuned to signaling traits used in intraspecific social interactions [26], species might exhibit the most tolerance toward humans wearing their sexually-selected signaling color(s) as suggested in previous studies of birds [18–20,25]. We conducted this study on western fence lizards (*Sceloporus occidentalis*) in Southern California. Male fence lizards have sexually-selected ventral blue patches on their abdomen and throat that they use in conspecific communication, displaying them through lateral flattening and/or a head-bobbing and push-up display. Although no studies have explicitly examined the role of these patches in sexual selection within *S*. *occidentalis*, work in congeners has demonstrated that males detect and respond to variation in ventral patch size, while females respond to variation in the throat patch but not the abdominal patch [27–29]. Thus, these ventral blue patches are important for intra- and intersexual selection in other *Sceloporus*.

Our extension of the species confidence hypothesis predicts that lizards should respond less fearfully to humans wearing their sexually-selected signaling color (blue) compared to other colors. We also tested whether individuals from a population living with high human activity differentiated less among colored T-shirts compared to lizards living with low human activity (i.e. effect of habituation). We selected T-shirts based on their color, and we accounted for their detectability against the background environment. If fence lizards have a bias to attend to the color blue, we expected them to tolerate closer approaches from a human wearing blue relative to other colors. If escape is primarily dependent on detectability (i.e. once detected, the lizard will escape to reduce costs associated with monitoring [30]), we expected fence lizards to exhibit enhanced escape responses towards humans wearing T-shirts that are more conspicuous. Finally, if lizards in the high-human activity site are habituated to humans, we expected them to exhibit reduced escape responses and also to discriminate less among T-shirt colors compared to those in the low-activity site.

## Materials and methods

### Study sites

We tested lizards at two sites in Southern California that vary in level of human activity. Griffith Park (34.134010°N, 118.288653°W, WGS84) is a heavily trafficked public park in Los Angeles, while Stunt Ranch (34.094491°N, 118.656699°W) is part of the University of California Natural Reserve System and is not accessible to the public. Both sites are within the Santa Monica Mountain Range and have similar habitats and background environments (mixed oak woodland, sage scrub, and chaparral). To quantitatively assess levels of human activity, we documented the number of people encountered per hour while performing behavioral trials at each site. Griffith Park had higher human activity than Stunt Ranch with 15 persons/hour compared to 2 persons/hour, respectively. All trials were conducted on weekdays so our estimate of the number of persons/hour at Griffith Park is conservative because park attendance increases on weekends.

### T-shirt colors

We selected two blue T-shirts that appeared to match the color of the lizards’ ventral patches, but differed in brightness to the human eye (dark blue and light blue). We also included a red and a gray T-shirt because both have reflectance spectra distinct from blue. Gray is also a background color on the lizards, but probably not a sexually-selected signaling color and should be less conspicuous than red. We used reflectance spectroscopy to measure the peak wavelengths and reflectance spectra of each T-shirt and of the blue ventral patches of adult male *S*. *occidentalis*. We caught and measured multiple body regions of four adult male *S*. *occidentalis* at Stunt Ranch: dorsal blue, dorsal background (brown/gray), center of the blue throat patch, throat background, black border of the blue abdominal patch on the left side, center of the blue abdominal patch on the left side, and abdomen background. For each area of interest, we took three separate measurements, which we averaged together. We used an Ocean Optics spectrometer (USB 2000) with a fiber optic reflectance probe (Ocean Optics R200-7-UV-VIS) and a pulsed xenon light source (Ocean Optics PX-2) to measure reflectance in a 1.3 mm diameter patch. To reduce glare, the probe was placed at a 45° angle relative to the surface being measured. We measured reflectance relative to a Labsphere certified reflectance standard in Ocean Optics’ software OOIBase32. We processed and plotted reflectance spectra using the R package *pavo* [31] (S1 Fig, S1 Table).

To determine quantitatively whether our blue T-shirts resembled the lizards’ ventral blue patches, we used Vorobyev and Osorio's [32] receptor noise model to calculate the just noticeable difference values (JNDs) for both chromatic and luminance (achromatic) contrasts between all T-shirts and the lizards’ blue patches. A JND value less than 1 indicates that two regions cannot be discriminated while a JND greater than 1 indicates that the regions are visually distinct, with higher values indicating greater contrast. We used the vismodel function in *pavo* to calculate the quantal catch from the visual system of the collared lizard *Crotaphytus dickersonae* (from [33]), using the D65 illuminant, and calculating the illuminance catch based on the sum of all cones. We then calculated JNDs from data generated from this visual model. Because published information on the retinal cone sensitivities of *Sceloporus* lizards is not available, we used the receptor spectral sensitivities of the collared lizard, another diurnal tetrachromatic iguanid lizard species, which has been used previously as a proxy for *Sceloporus* vision [34]. We used the default values for Weber fractions and cone ratios in the *pavo* package.

To determine the conspicuousness of the T-shirts in the environment, we took photographs of one person (BJP) wearing each T-shirt at 10 randomly selected locations in each study site. Photos were taken at a distance of 2 m from the observer who was wearing the T-shirt along with an 18% gray standard that was centered in the camera’s field of view. Pictures were taken with a Canon Rebel XSi fitted with an EF-S 18–55 mm lens (Canon U.S.A., Inc; Melville, NY); we used manual settings for shutter speed and lens aperture, the white balance was set to daylight, and images were saved in RAW file format. All photos were taken with the observer in the open, in direct sunlight, and facing the sun to minimize differences in lighting among images. Photos were taken between 0900–1300 h, the same timeframe when behavioral trials occurred.

We used the Multispectral Image Calibration and Analysis Toolbox plugin for ImageJ [35] to analyze our images. Prior to analysis, images were screened for exposure issues and we found that several had been overexposed. Thus only 4 from Stunt and 6 from Griffith Park were used. Suitable images were linearized and equalized using the gray standard. We transformed the camera’s red, green, and blue channel pixel values to a lizard-specific color space, converting to animal cone-catch values using the camera spectral sensitivity functions (under the D65 illuminant), database of natural spectra (400–700 nm), and the visual system of the collared lizard. In short, simulations predict the photoreceptor responses of the lizard to the natural spectra, and the camera's responses to the same spectra under the D65 standard illuminant. A model is then generated that can predict animal photoreceptor cone-catch values from camera values.

For each pixel, we calculated long wave sensitive (LWS), medium wave sensitive (MWS), short wave sensitive (SWS), and double cone photon catches (LUM) of the model species. Although fence lizards likely are able to detect UV, we regarded that the UV cone type would play a negligible role in the relative differences in detectability among the shirts because the shirts reflected minimally in the UV range (S1 Fig). We divided each image into 2 regions: T-shirt (area just below the gray standard) and background (all area surrounding the observer from the waist up). We manually selected these regions using the ‘rectangular selection’ and ‘polygon selection’ tools in ImageJ respectively, and we calculated JNDs between these two regions for color and luminance, thus accounting for chromatic and achromatic contrasts respectively [36,37]. We used a Weber fraction of 0.05, and we assumed equal cone ratios (following [34,38]). With these comparisons, we could determine whether lizards were responding to color *per se* or conspicuousness.

### Behavioral trials

Methods were approved by the CSU Northridge Institutional Animal Care and Use Committee (1516-002b). We conducted behavioral trials from 18 August to 11 September 2016 between 0900–1300 h. We measured flight-initiation distance (FID) and the probability of capture post-FID trial (y/n). FID is the distance at which an animal flees from an approaching threat, and is a measure of risk assessment and of an animal’s tolerance of humans [39]. Longer FIDs correspond to an increased perception of risk in animals [40]. To quantify FID, the observer walked around slowly looking for lizards that were active (i.e., foraging, basking, or alert on a perch) but not running or walking. Once spotted, the observer walked directly toward the lizard (pace of 0.5 m/s, which was standardized beforehand) and noted when it fled (any movement away from the initial location) and the total distance between the lizard and the observer when the trial was started (termed the start distance). FID was estimated as the Euclidian distance between the observer and the lizard, which equals the square root of the sum of the squared horizontal distance and the squared height of the lizard above ground level (quantified using measuring tape after the trial), thus accounting for varied heights among sampled individuals [41].

Three days into the study, the observer started capturing lizards to obtain data on sex and morphometric measurements. After an FID trial, the observer attempted to capture the lizard with a noose tied to the end of a fishing pole, a standard technique for catching lizards. The person pursued the lizard until it was captured or it sought an unreachable location (e.g. it ran into a refuge or too far away). Lizards that did not flee long distances and/or into a refuge during the FID trial were more likely to be captured. The distance lizards were from the closest refuge when they were initially located was also recorded because this could influence FID [11]. If the lizard fled to refuge during the trial, the distance from its initial location to its hiding place was measured. However, if the lizard did not seek refuge, the distance to the nearest crevice/hole that a lizard could hide in or vegetation that could conceal the lizard was measured.

One person performed all fieldwork (BJP). Sites were visited haphazardly to avoid sampling order effects. T-shirts were worn in random order and the color was changed every 3–4 trials per day. Ambient temperatures do not affect FID [11], but we still only conducted trials on warm, sunny days (> 22°C). Because T-shirts were worn in a random order each day, temperature, which is correlated with time of day, is unlikely to affect the results in some systematic way. Only lizards that were relatively large adults were sampled, and we did not exclude lizards with autotomized tails because this does not affect escape behavior [11]. We avoided repeating trials on the same individuals by continually walking in one direction and not sampling the same location twice. All lizards that were captured were also visibly marked with non-toxic paint to ensure these individuals were not re-sampled. The observer always wore the same base layer of clothing consisting of a black tank top (underneath the colored T-shirt), black pants, and light brown hiking boots. Hair was always pulled back into a ponytail, and sunscreen was used on the face, neck, and arms (Coppertone® Sport SPF 50). Clothing was washed three times during this study, only when it had become noticeably dirty. Clothes were hand washed using Dr. Bronner’s unscented baby soap and hung to dry.

### Statistical analyses

All analyses were done in R (R v3.2.1, R Development Core Team, 2015). We used descriptive statistics of JND values to evaluate which T-shirts most resembled the lizards’ ventral blue patches and which T-shirts were most conspicuous against the background environment, and we performed ANOVAs to determine statistical differences in mean JNDs among the T-shirts. We fitted generalized linear models (GLMs) to examine variation in escape behavior. Our *a priori* hypothesis was that lizards’ FID would be sensitive to T-shirt color. However, at the end of the study, we noticed that the probability of capture seemed to also associate with T-shirt color and so we included this as another response variable. A higher probability of capture was typically associated with reduced escape behavior. Because the observer had no *a priori* assumption that capture success would be affected by color, and because one person performed all fieldwork, bias and variation among treatments should be minimized.

We tested for sex differences and found no effect of sex on FID (t_89_ = 0.345, p = 0.731) or on proportion captured (*χ*^2^ = 0.526, p = 0.468). Because sex did not affect the two response variables, we did not include it in further analyses. In our GLM on FID, we included the main factors of T-shirt color and site, the covariates of start distance and distance to refuge, and the interaction between color and site to examine whether human activity influences response to color. We square-root transformed FID to meet model assumptions. For probability of capture post-trial, we fitted a binomial GLM with color and site as independent variables and used the Wald test to estimate p-values. We could not examine the color*site interaction for probability of capture because we did not start capturing lizards until three days into the study, limiting our sample size from Griffith Park. We used the *compute*.*es* package [42] in R to calculate effect sizes (Cohen’s d) among treatments and the *lsmeans* package [43] to run post-hoc comparisons. We corrected for multiple comparisons among T-shirt colors using the false discovery rate, and we set our alpha to 0.05.

## Results

### T-shirt colors

Quantitatively, the two blue T-shirts were more like the lizards’ throat and abdominal blue patches (i.e., they had the lowest JNDs) than the gray or red shirt for chromatic (throat: F = 9.28, p = 0.006; abdomen: F = 3.99, p = 0.035) and luminance (throat: F = 7.60, p = 0.010; abdomen: F = 17.10, p < 0.001) contrasts. The light blue T-shirt had the lowest mean chromatic JND when compared with the throat patch, while both light blue and dark blue shirts had the lowest chromatic JNDs when compared with the abdominal patch (Table 1). However, gray was not statistically different from the blue shirts in terms of chromatic contrast (S2 Table). In terms of luminance, dark blue had the lowest mean JND when compared to the throat patch, while light blue had the lowest mean JND when compared to the abdominal patch (Table 1). The gray and red shirts were statistically more distinguishable from the lizard blue patches compared to the blue shirts (S2 Table, S3 Table).

**Table 1:** Mean ± SD of JNDs for chromatic and luminance (achromatic) contrasts. To determine whether T-shirts differed in their resemblance to the sexually-selected blue patches of male *Sceloporus occidentalis*, we calculated JNDs by comparing T-shirts to the throat patch and to the abdominal patch of lizards. To determine whether T-shirts differed in their detectability, we calculated JNDs by comparing T-shirts to the background environment from photographs taken within each study site.

Contrast type	Comparison region	T-shirt color			
		Dark blue	Light blue	Gray	Red
Chromatic	Throat	3.0 ± 1.5	2.3 ± 1.0	3.4 ± 1.8	8.2 ± 1.7
	Abdomen	7.2 ± 1.2	7.2 ± 1.3	7.6 ± 1.5	9.8 ± 1.1
	Background environment	10.3 ± 3.4	6.9 ± 3.2	3.6 ± 1.0	59.2 ± 6.4
Luminance	Throat	1.4 ± 1.2	3.6 ± 1.7	7.5 ± 1.7	5.1 ± 1.7
	Abdomen	5.2 ± 2.4	2.0 ± 1.0	11.7 ± 2.4	9.3 ± 2.4
	Background environment	7.6 ± 6.8	16.6 ± 10.5	13.9 ± 8.1	8.2 ± 7.1

The four T-shirts also differed in conspicuousness against the background environment for chromatic (F = 314.6, p < 0.001) and luminance (F = 4.98, p = 0.004) contrasts and all pairwise comparisons were significant for the chromatic contrasts with red being the most conspicuous followed by dark blue, light blue, and gray (Table 1, S4 Table). In terms of luminance, light blue was the most contrasting, followed by gray, red, and dark blue (Table 1). Statistical pairwise comparisons can be found in S4 Table.

### Behavioral trials

**Fig 1:**
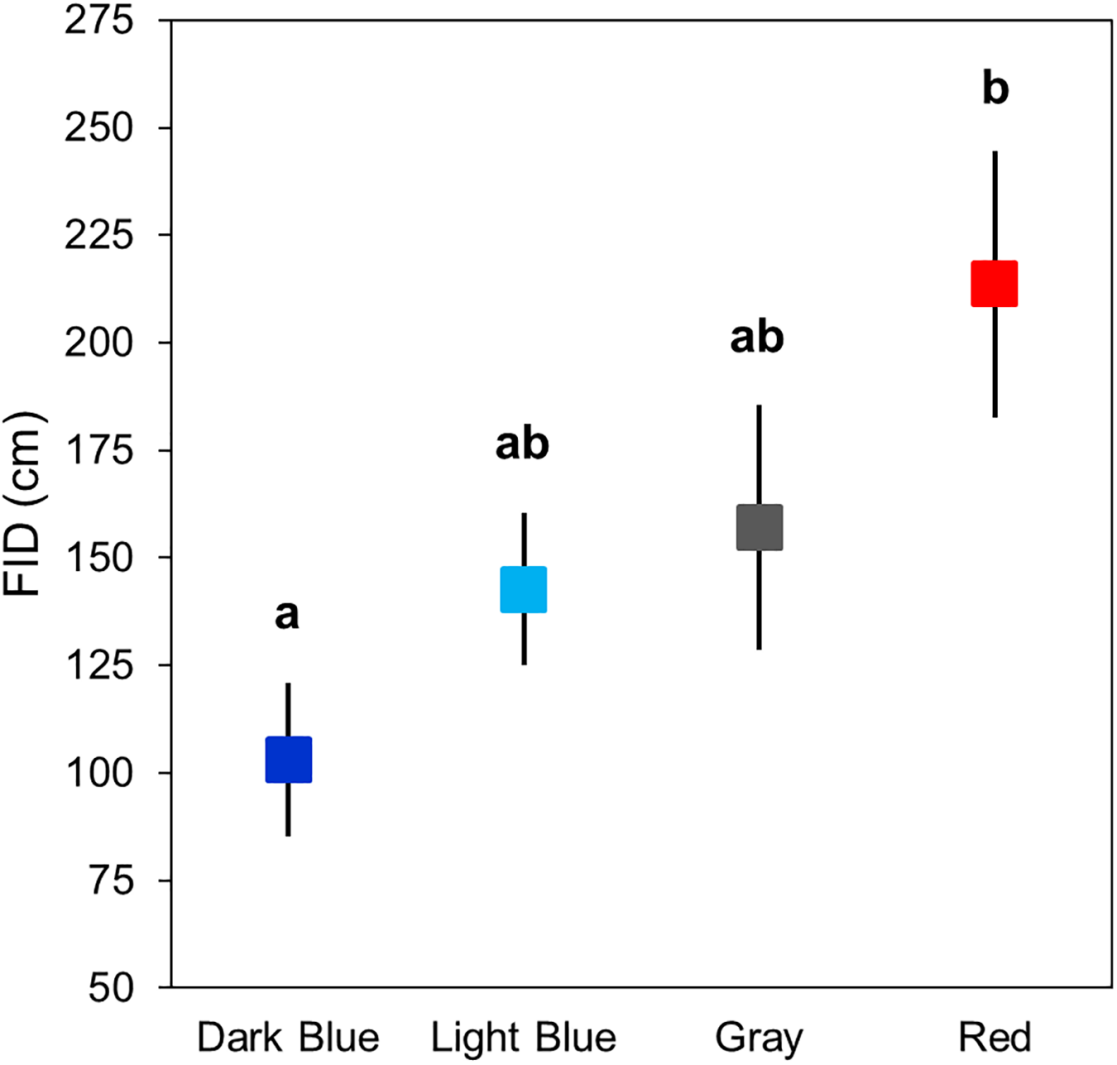
Mean ± SE flight initiation distance (FID) of lizards approached by a human wearing different colored T-shirts (data from both sites pooled). Letters denote significant (p < 0.05) differences among colors from post-hoc comparisons made using the false discovery rate.

**Fig 2:**
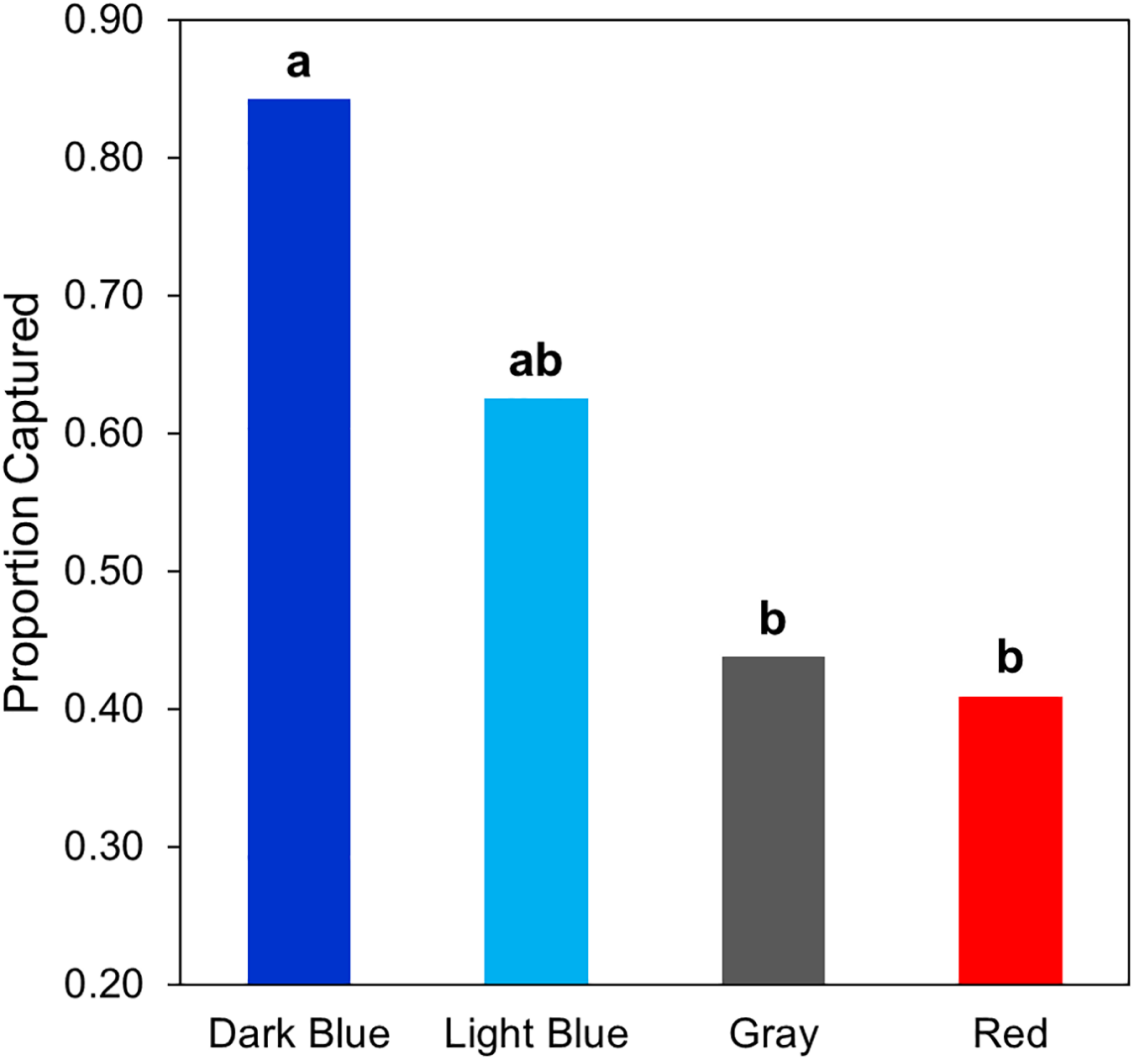
Proportion of lizards that were captured after the FID trial for each color of T-shirt (data from both sites pooled). Letters denote significant (p < 0.05) differences among colors from post-hoc comparisons made using the false discovery rate.

We conducted 15 dark blue, 14 light blue, 15 gray, and 15 red trials in Griffith Park and 14 dark blue, 14 light blue, 13 gray, and 14 red trials in Stunt Ranch. T-shirt color influenced the distance at which lizards fled from an approaching threat (F = 2.89, p = 0.039, Fig 1) and probability of capture (*χ*^*2*^ = 8.32, p = 0.040, Fig 2), but level of human activity did not affect either behavior (FID: F = 1.51, p = 0.222; capture: *χ*^*2*^ = 1.17, p = 0.280). After explaining significant variation in FID by start distance (F = 5.23, p = 0.024), and non-significant variation by distance to refuge (F = 1.83, p = 0.179), the effect of color on FID was not influenced by level of human activity (color*site: F = 0.154, p = 0.927).

After correcting for multiple comparisons, we found that lizards fled at longer distances when approached with red compared to dark blue (Fig 1), a difference with a large effect (p = 0.033, Cohen’s d = 0.83). FIDs were also nearly significantly different between trials with light blue and red T-shirts (p = 0.082, Cohen’s d = 0.68). All other comparisons failed to reach statistical significance and had smaller effect sizes (all p > 0.10 and Cohen’s d < 0.50). Lizards were captured 84% of the time when wearing dark blue, but this rate dropped by nearly half with gray or red (Fig 2). The differences in capture rates between dark blue and gray, and between dark blue and red were significant and had large effect sizes (both p = 0.048 and Cohen’s d = 0.87). Probability of capture while wearing light blue did not differ from the other colors and these comparisons had smaller effect sizes (Cohen’s d = 0.30–0.50). There was nearly no difference in capture rates between red and gray (p = 0.939, Cohen’s d = 0.0).

## Discussion

We found support for a prediction of our modified version of the species confidence hypothesis; FID was lower and capture rate was higher during trials in which a human observer was wearing a T-shirt that resembled the sexually-selected signaling color of the test species, in this case, western fence lizards. These responses were the same across two sites that differed in level of human activity suggesting that habituation has not occurred or is not important in influencing these escape behaviors.

The differences in FID that we recorded could potentially result from red being the most conspicuous color in the environment (based on color JND scores), and animals should flee immediately or soon after detecting an approaching threat because of the costs associated with monitoring [30,39]. Red is not found on, and therefore should not be associated with, any of the lizards’ predators, but it could also represent a novel, threatening stimulus because this color is not common in the natural environment. However, gray elicited behavioral responses similar to red, even though it should be perceived differently than red. Differences in capture rate are likely due to the specific color of T-shirt and not its detectability because gray, which affected capture similarly as red (44% and 41% respectively), had lower color (chromatic) contrast than dark blue for which the capture rate was 84%. Furthermore, dark blue was no different, in terms of luminance contrast, from red, suggesting that these two colors should have elicited similar capture rates if lizards were responding to detectability based on achromatic contrast. Regardless of how the lizards perceive these colors, our results demonstrate that a T-shirt that appears dull to the human eye (i.e. gray) does not necessarily have the least behavioral effect on the test animal. Studies using FID to measure animal risk assessment often employ approaches with a human wearing dull or neutral colored clothes (see [17,44,45]). This method can be appropriate for studies investigating relative differences in behavior between treatments if the observer always wears the same outfit. However, dull or neutral clothing may not be the most appropriate choice if humans wish to have the least disturbance and/or lowest FID on animals, such as in the context of ecotourism.

This is the first study to demonstrate that a lizard species, like some bird species [18–20], respond differently to observer clothing colors and do so in a manner consistent with the species confidence hypothesis. Reduced escape occurred most often under the dark blue treatment, facilitated easy capture of lizards, and suggests that they might have a preferential bias toward this color [46]. It will be worthwhile to test other species (of lizards and other taxa) with different signaling colors and species that lack sexually-selected body coloration to determine whether our results are part of a more general pattern in nature. Further studies should also attempt to assess the underlying mechanism driving this pattern. Although a sensory bias should improve detection ability [26], in the case of potential predators, it is not clear why once detected the prey individual allows the threat to get closer than if it lacked the sexually-selected signal color. Typically, costs of monitoring are assumed to be high [30], but if the color indicates a potential territorial intruder or mate, it is possible that the costs of not attending could be higher because of missed opportunities to enhance reproductive success. Irrespective of the mechanism, our results reveal important implications for human impacts on wildlife that we discuss below.

First, choice of clothing can influence research outcomes for biologists working with free-ranging animals; behavioral results (i.e. FID values) and hand-capture rates were both influenced by T-shirt color. Lizards were nearly twice as likely to be captured when the observer was wearing a dark blue shirt relative to red or gray. Hence, the choice of clothing worn in the field could impact a researcher’s time and budget if animals are unable to be captured effectively. Additionally, if researchers are studying animal behavior in the field, their results could be influenced by their choice of clothing colors. Clearly in some cases, such as for goat-antelope (*R*. *rupicapra*) [21], behavior will not be strongly affected by color. However, as a precaution, we recommend either to standardize or to randomize clothing when designing and conducting field research, depending upon desired inferences. Pilot studies could also be done to determine which type of clothing is likely to enhance research efficiency and effectiveness.

Clothing color could also be important from an ecotourism and conservation standpoint. If humans wish to visit natural areas to view animals and create the least disturbance while doing so, wearing certain colors could better achieve this goal. Based on the few available studies, it appears that ecotourists hoping to see a particular species should wear colors found on that species, and ideally colors involved in signaling to conspecifics, although the generality of this assumption requires further testing. In addition, long flight distances and exaggerated escape responses could have fitness consequences for animals if they significantly alter their activity budgets. For instance, lizards that fled to refuge or far away could exhibit reductions in foraging time if their hiding times are lengthy. Because we do not know whether the escape behaviors we recorded reduce body condition (due to reduced foraging and physiological stress as in [47,48], future work is necessary to determine the fitness effects for this and other species.

Finally, we found no differences in lizard escape responses between an area of high human activity and low human activity. There are three potential explanations for this result: 1) repeated encounters with humans do not lead to habituation in this species; 2) the encounter rates with humans at the high-visitation site (Griffith Park) were not high enough to cause a reduction in escape behaviors; or 3) the encounter rates at the low-visitation location (Stunt Ranch) were high enough to cause habituation. More work will be required to determine which levels of human activity, and the duration of time experiencing them, are necessary for animals to become habituated or sensitized to human disturbances [16].

## Supporting information

S1 Table Descriptions of color measurements of the T-shirts and of four lizard body regions. T-shirt colors were chosen based on whether their peak wavelengths were similar to those of the sexually-selected signaling color patches of adult male *Sceloporus occidentalis*. For comparison, two non-signaling body regions are also shown.
(DOCX)

S2 Table Results of post-hoc comparisons from the ANOVAs comparing the mean chromatic and luminance JNDs of each T-shirt color to the lizards’ blue throat patch. P values were corrected for multiple comparisons using the false discovery rate. Contrasts that met statistical significance (p < 0.05) are in bold.
(DOCX)

S3 Table Results of post-hoc comparisons from the ANOVAs comparing the mean chromatic and luminance JNDs of each T-shirt color to the lizards’ blue abdominal patch. P values were corrected for multiple comparisons using the false discovery rate. Contrasts that met statistical significance (p < 0.05) are in bold.
(DOCX)

S4 Table Results of post-hoc comparisons from the ANOVAs comparing the mean chromatic and luminance JNDs of each T-shirt color to the background environment. P values were corrected for multiple comparisons using the false discovery rate. Contrasts that met statistical significance (p < 0.05) are in bold.
(DOCX)

S1 Fig Reflectance spectra plots of the four colored T-shirts and various lizard body regions. (PDF)
