# Supplementary material for: Fear no colors? Observer clothing color influences lizard escape behavior
Source: PLoS One. 2017 Aug 9;12(8):e0182146. doi: 10.1371/journal.pone.0182146 (PMC5549895; doi:10.1371/journal.pone.0182146)
Supplement: S1 Table — T-shirt colors were chosen based on whether their peak wavelengths were similar to those of the sexually-selected signaling color patches of adult male Sceloporus occidentalis. For comparison, two non-signaling body regions are also shown. (DOCX) [file pone.0182146.s001.docx]

**S1 Table. Descriptions of color measurements of the T-shirts and of four lizard body regions.** T-shirt colors were chosen based on whether their peak wavelengths were similar to those of the sexually-selected signaling color patches of adult male *Sceloporus occidentalis*. For comparison, two non-signaling body regions are also shown.

| Object measured | Signal color | Wavelength of peak reflectance | Intensity of peak reflectance |
| --- | --- | --- | --- |
| Dark blue shirt | Yes | 451 | 21.0 |
| Light blue shirt | Yes | 461 | 26.2 |
| Gray shirt | No | 700 | 17.6 |
| Red shirt | No | 689 | 39.7 |
| Lizard abdominal patch | Yes | 500 | 29.4 |
| Lizard throat patch | Yes | 488 | 19.2 |
| Lizard dorsal background | No | 641 | 8.4 |
| Lizard ventral background | No | 693 | 18.8 |
