# Supplementary material for: Fear no colors? Observer clothing color influences lizard escape behavior
Source: PLoS One. 2017 Aug 9;12(8):e0182146. doi: 10.1371/journal.pone.0182146 (PMC5549895; doi:10.1371/journal.pone.0182146)
Supplement: S2 Table — P values were corrected for multiple comparisons using the false discovery rate. Contrasts that met statistical significance (p < 0.05) are in bold. (DOCX) [file pone.0182146.s002.docx]

**S2 Table. Results of post-hoc comparisons from the ANOVAs comparing the mean chromatic and luminance JNDs of each T-shirt color to the lizards’ blue throat patch.** P values were corrected for multiple comparisons using the false discovery rate. Contrasts that met statistical significance (p < 0.05) are in bold.

|  | Chromatic JNDs | | | Luminance JNDs | | |
| --- | --- | --- | --- | --- | --- | --- |
| JND contrast | t ratio | df | p value | t ratio | df | p value |
| Dark blue–Gray | -0.38 | 8 | 0.715 | -4.62 | 8 | **0.010** |
| Dark blue–Light blue | 0.51 | 8 | 0.715 | -1.64 | 8 | 0.168 |
| Dark blue–Red | -4.20 | 8 | **0.009** | -2.83 | 8 | **0.044** |
| Gray–Light blue | 0.89 | 8 | 0.603 | 2.99 | 8 | **0.044** |
| Gray–Red | -3.82 | 8 | **0.010** | 1.79 | 8 | 0.167 |
| Light Blue–Red | 4.71 | 8 | **0.009** | -1.20 | 8 | 0.266 |
