# Supplementary material for: Fear no colors? Observer clothing color influences lizard escape behavior
Source: PLoS One. 2017 Aug 9;12(8):e0182146. doi: 10.1371/journal.pone.0182146 (PMC5549895; doi:10.1371/journal.pone.0182146)
Supplement: S3 Table — P values were corrected for multiple comparisons using the false discovery rate. Contrasts that met statistical significance (p < 0.05) are in bold. (DOCX) [file pone.0182146.s003.docx]

**S3 Table. Results of post-hoc comparisons from the ANOVAs comparing the mean chromatic and luminance JNDs of each T-shirt color to the lizards’ blue abdominal patch.** P values were corrected for multiple comparisons using the false discovery rate. Contrasts that met statistical significance (p < 0.05) are in bold.

|  | Chromatic JNDs | | | Luminance JNDs | | |
| --- | --- | --- | --- | --- | --- | --- |
| JND contrast | t ratio | df | p value | t ratio | df | p value |
| Dark blue–Gray | -0.48 | 12 | 0.793 | -3.83 | 12 | **0.005** |
| Dark blue–Light blue | -0.03 | 12 | 0.978 | 2.74 | 12 | **0.026** |
| Dark blue–Red | -2.96 | 12 | **0.038** | -2.64 | 12 | **0.026** |
| Gray–Light blue | 0.45 | 12 | 0.793 | 6.57 | 12 | **< 0.001** |
| Gray–Red | -2.48 | 12 | 0.058 | 1.20 | 12 | 0.254 |
| Light Blue–Red | -2.93 | 12 | **0.038** | -5.37 | 12 | **0.001** |
