# Supplementary material for: Fear no colors? Observer clothing color influences lizard escape behavior
Source: PLoS One. 2017 Aug 9;12(8):e0182146. doi: 10.1371/journal.pone.0182146 (PMC5549895; doi:10.1371/journal.pone.0182146)
Supplement: S4 Table — P values were corrected for multiple comparisons using the false discovery rate. Contrasts that met statistical significance (p < 0.05) are in bold. (DOCX) [file pone.0182146.s004.docx]

**S4 Table. Results of post-hoc comparisons from the ANOVAs comparing the mean chromatic and luminance JNDs of each T-shirt color to the background environment.** P values were corrected for multiple comparisons using the false discovery rate. Contrasts that met statistical significance (p < 0.05) are in bold.

|  | Chromatic JNDs | | | Luminance JNDs | | |
| --- | --- | --- | --- | --- | --- | --- |
| JND contrast | t ratio | df | p value | t ratio | df | p value |
| Dark blue–Gray | 8.38 | 60 | **< 0.001** | -2.34 | 66 | **0.044** |
| Dark blue–Light blue | 3.77 | 60 | **< 0.001** | -3.21 | 66 | **0.012** |
| Dark blue–Red | -21.74 | 60 | **< 0.001** | -0.23 | 66 | 0.822 |
| Gray–Light blue | -4.35 | 60 | **< 0.001** | -0.94 | 66 | 0.422 |
| Gray–Red | -29.23 | 60 | **< 0.001** | 2.12 | 66 | 0.057 |
| Light Blue–Red | -24.61 | 60 | **< 0.001** | 2.99 | 66 | **0.012** |
