# Supplementary material for: Fear no colors? Observer clothing color influences lizard escape behavior
Source: PLoS One. 2017 Aug 9;12(8):e0182146. doi: 10.1371/journal.pone.0182146 (PMC5549895; doi:10.1371/journal.pone.0182146)
Supplement: S1 Fig — (PDF) [file pone.0182146.s005.pdf]

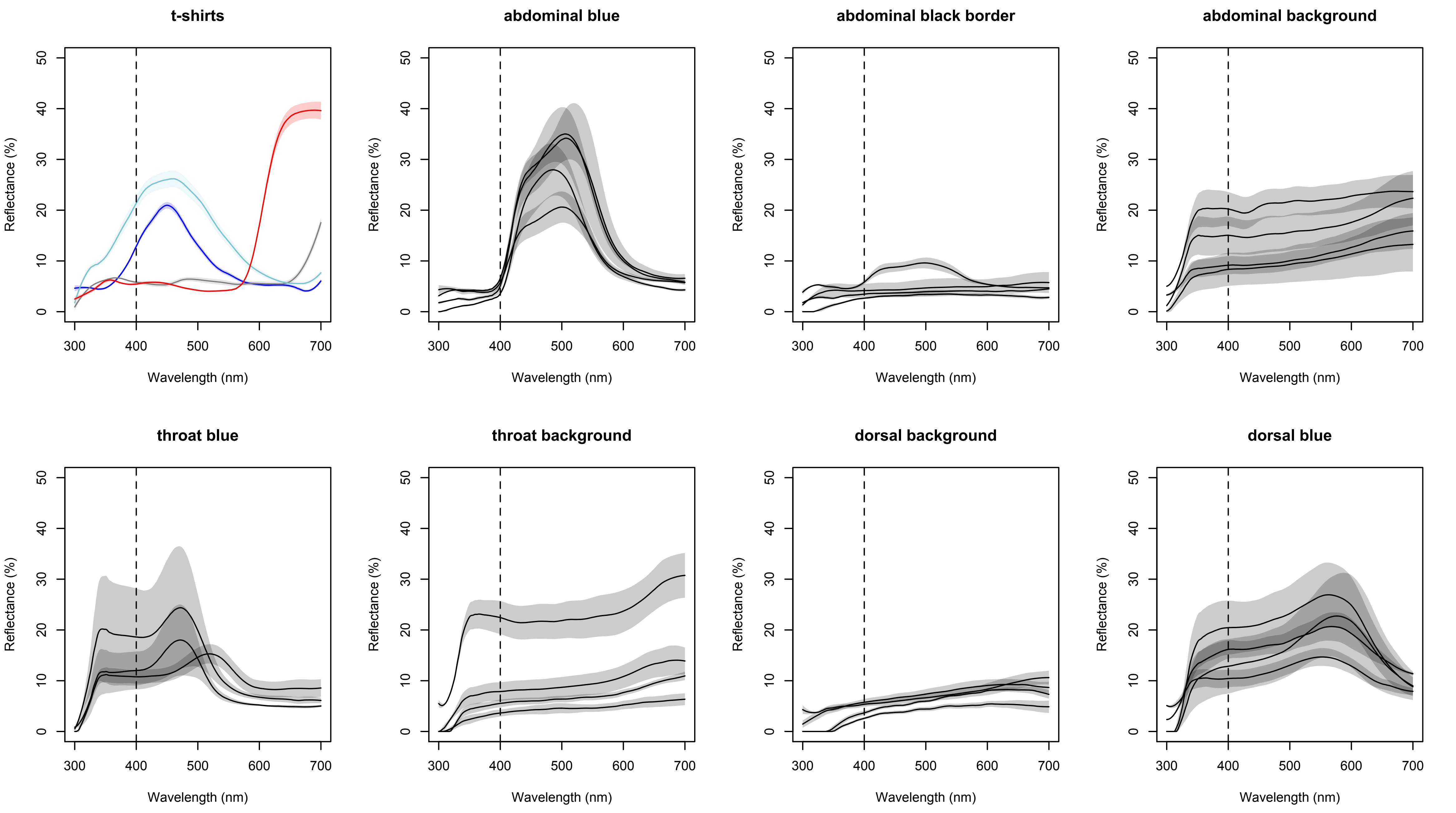
t-shirts
abdominal blue
abdominal black border
abdominal background
throat blue
throat background
dorsal background
dorsal blue
Reflectance (%)
Wavelength (nm)
0
10
20
30
40
50
300
400
500
600
700
